# The Impact of the COVID-19 Pandemic on Kidney Transplant Candidate Waitlist Status across Demographic and Geographic Groups: A National Analysis of UNOS STAR Data

**DOI:** 10.3390/healthcare11040612

**Published:** 2023-02-18

**Authors:** Conner V. Lombardi, Jacob J. Lang, Meng-Hao Li, Abu Bakkar Siddique, Naoru Koizumi, Obi Ekwenna

**Affiliations:** 1 Department of Urology and Transplantation, University of Toledo College of Medicine and Life Sciences, Toledo, OH 43614, USA; 2 Schar School of Policy and Government, George Mason University, Fairfax, VA 22030, USA

**Keywords:** COVID-19, transplant, kidney transplant, UNOS, pandmeic

## Abstract

The primary goal of this retrospective study is to understand how the COVID-19 pandemic differentially impacted transplant status across race, sex, age, primary insurance, and geographic regions by examining which candidates: (i) remained on the waitlist, (ii) received transplants, or (iii) were removed from the waitlist due to severe sickness or death on a national level. **Methods:** The trend analysis aggregated by monthly transplant data from 1 December 2019 to 31 May 2021 (18 months) at the transplant center level. Ten variables about every transplant candidate were extracted from UNOS standard transplant analysis and research (STAR) data and analyzed. Characteristics of demographical groups were analyzed bivariately using t-test or Mann–Whitney U test for continuous variables and using Chi-sq/Fishers exact tests for categorical variables. **Results:** The trend analysis with the study period of 18 months included 31,336 transplants across 327 transplant centers. Patients experienced a longer waiting time when their registration centers in a county where high numbers of COVID-19 deaths were observed (SHR < 0.9999, *p* < 0.01). White candidates had a more significant transplant rate reduction than minority candidates (−32.19% vs. −20.15%) while minority candidates were found to have a higher waitlist removal rate than White candidates (9.23% vs. 9.45%). Compared to minority patients, White candidates’ sub-distribution hazard ratio of the transplant waiting time was reduced by 55% during the pandemic period. Candidates in the Northwest United States had a more significant reduction in the transplant rate and a greater increase in the removal rate during the pandemic period. **Conclusions:** Based on this study, waitlist status and disposition varied significantly based on patient sociodemographic factors. During the pandemic period, minority patients, those with public insurance, older patients, and those in counties with high numbers of COVID-19 deaths experienced longer wait times. In contrast, older, White, male, Medicare, and high CPRA patients had a statistically significant higher risk of waitlist removal due to severe sickness or death. The results of this study should be considered carefully as we approach a reopening world post-COVID-19, and further studies should be conducted to elucidate the relationship between transplant candidate sociodemographic status and medical outcomes during this era.

## 1. Introduction

The COVID-19 pandemic has had a substantial impact on access to kidney transplantation worldwide. From the onset of the pandemic, public health mitigation strategies, including delay of elective surgeries and staffing shortages compromised the volume of kidney transplant centers nationwide [1,2]. This was compounded with the deleterious effects of COVID-19 infection in patients awaiting and undergoing kidney transplants, as many of these patients are significantly immunocompromised. The pandemic’s impact has been multifaceted and has ultimately led to increases in kidney transplant waitlist mortality rates as well as decreases in deceased donor kidney transplantation (DDKT) and living donor kidney transplantation (LDKT) rates in the early stages of the pandemic [3,4,5,6]. While the COVID-19 pandemic has had these well-documented impacts on kidney transplantation rates and waitlist statuses, there is an existing research gap in how this impact has affected candidates’ waitlist status based on their demographic and geographic backgrounds.

Based on the literature currently available, disparities in access and outcomes for kidney transplantation among minority, publicly insured, and lower-income individuals are well-documented [7,8]. The impact of the early pandemic on kidney transplantation has only exacerbated these disparities. In a national cross-sectional analysis of patients with incident kidney failure via renal management information system medical evidence form during the first four months of the pandemic, Black patients and patients in counties with significantly higher COVID-19 mortality rates had lower rates of initiation of treatment [9]. A recent study of the Scientific Registry of Transplant Recipients (SRTR) database, which includes publicly available information on donors, waitlist candidates, and transplant recipients in the United States, found that Black patients, as well as those with public insurance, experienced significantly lower counts of LDKT compared to White individuals in the first 12 months of the pandemic [10].

However, the extent to which COVID-19 has impacted patients across sociodemographic groups, including sex, age, primary insurance, center transplant volume, and geographic region in addition to race on the patient waitlist status, has not been fully elucidated. The primary goal of this retrospective study is to understand how the COVID-19 pandemic differentially impacts transplant status across the aforementioned sociodemographic groups. We have proposed the following research questions pertaining to the COVID-19 pandemic and sociodemographic considerations of transplant candidates: Which transplant candidates remained on the waitlist? Which candidates received transplants? Which transplant candidates were removed from the waitlist due to severe sickness or death?

## 2. Methods

### 2.1. Data

For the investigation, this study combined the two large-scale national level statistics, i.e., kidney transplant registry data from the United Network for Organ Sharing (UNOS) standard transplant analysis and research (STAR) file and the census data. UNOS manages the organ transplant system under contract with the U.S. federal government and collects and reports data on every U.S. organ donor, transplant candidate, recipient, and outcome.

### 2.2. Statistical Analytic Plan

**Figure 1:**
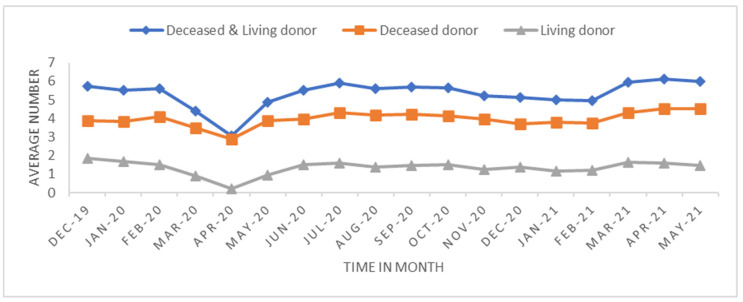
Average transplant by donor type.

**Figure 2:**
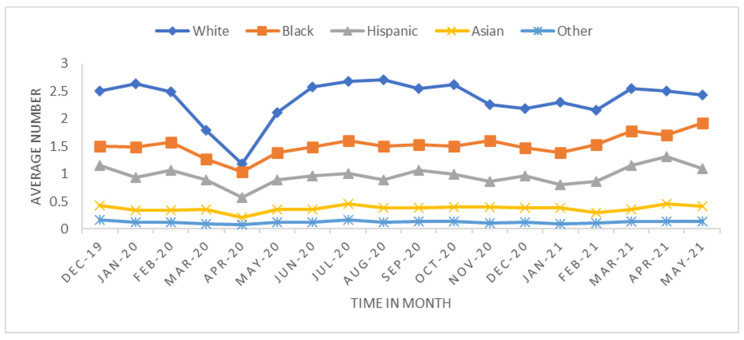
Average overall transplant rate by race.

**Figure 3:**
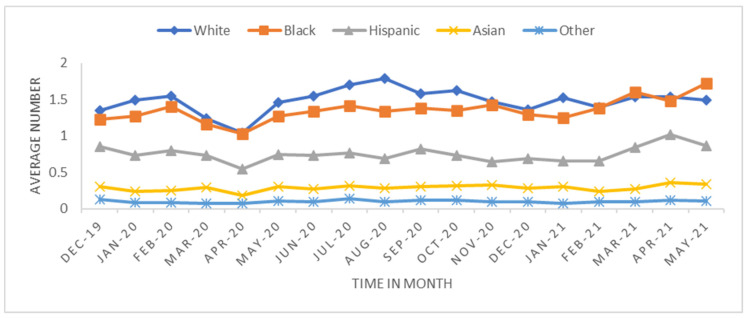
Average deceased donor transplant rate by race.

**Figure 4:**
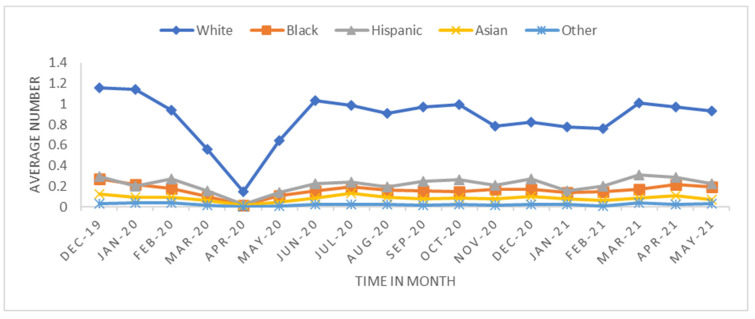
Average living donor transplant by race.

**Figure 5:**
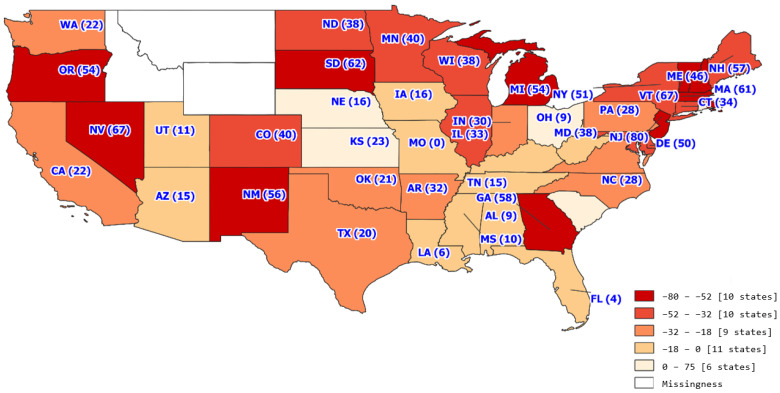
Transplant change rate by state.

**Figure 6:**
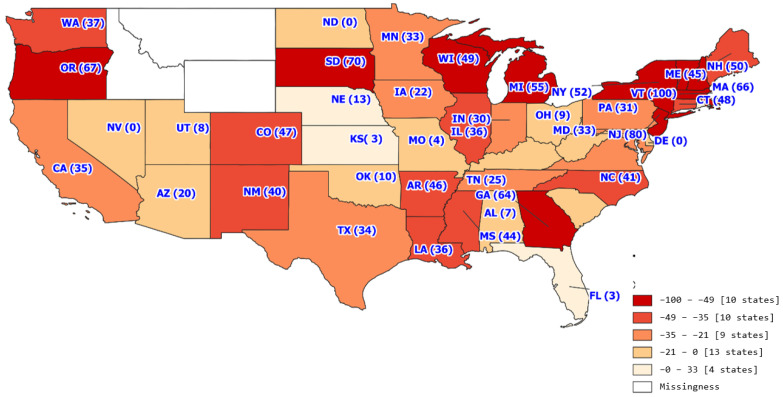
White transplant change rate by state.

**Figure 7:**
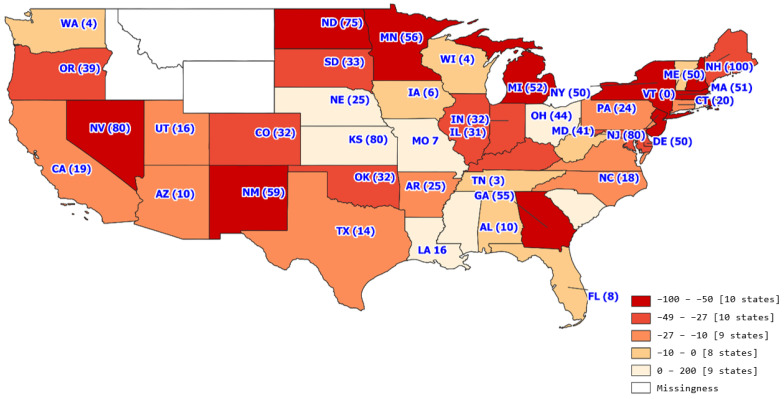
Minority transplant change rate by state.

We performed two analyses: One looked at the overall trend, and the other measured the short-term effect in the early stage of the pandemic. The trend analysis was aggregated by monthly transplant data from 1 December 2019 to 31 May 2021 (18 months) at the transplant center level. The monthly average transplant rates were calculated to investigate how the transplant rate changed before and after the pandemic began (Figure 1, Figure 2, Figure 3 and Figure 4). The short-term effect analysis restricts the study period to six months (December 2019–May 2020): the pre-pandemic (December 2019–February 2020) and the early pandemic (March 2020–May 2020). The short-term effect analysis aimed to investigate how the COVID-19 pandemic impacted different race groups in accessing transplants before and during the pandemic period (Table 1, Table 2, Table 3, Table 4 and Table 5 and Figure 5, Figure 6 and Figure 7). Candidate sex, age, race, primary insurance, center transplant volume, and geographic regions, donor status, waitlist status, calculated panel reactive antibodies (CPRA), and kidney donor profile index (KDPI) were extracted from the STAR data and analyzed. The CPRA value is used to determine the incompatibility between the candidate and donor. The kidney allocation system will prioritize candidates with a CPRA value of 80% or higher. KDPI is a combined donor index utilized to calculate the likelihood of graft failure after a deceased donor kidney transplant.

Candidate characteristics of the White and minority groups were compared bivariately using t-test for continuous variables and using Chi-square tests for categorical variables. The change in the transplant candidate status before and during the pandemic periods were calculated to examine the associations between demographic variables and the candidate status. We also applied the competing risk analysis to examine how race was associated with candidates’ time of receiving transplants or removal from the waitlist due to severe sickness or death. The competing risk analysis is a type of survival analysis used to tackle the violation of the independent censoring assumption in traditional survival analysis. For example, patients may experience more than one mutually exclusive event, such as time on the waitlist until kidney transplant or death. The rate of death depends on the rate of kidney transplants, and vice versa. With this condition, the competing risk analysis is commonly used to investigate the event of interest and preclude the occurrence of other competing events. In this study, severe sickness or death was the competing event when we analyzed the factors that influenced the waitlist time to kidney transplants. Similarly, when we examined the factors influencing the waitlist time to severe sickness or death, kidney transplant was the competing event.

All statistical analyses were performed using STATA (v. 14, Stata Corp), and statistical significance was defined as the 5% level unless noted otherwise.

## 3. Results

### 3.1. Descriptive Statistics

The trend analysis with the study period of 18 months included 31,336 transplants across 327 transplant centers. The average number of transplants per month per transplant center was 5.32 with a standard deviation of 7.76. Figure 1, Figure 2, Figure 3 and Figure 4 show the average number of transplants by month at the transplant center level. It shows that the transplants number dropped by a large margin immediately during the early COVID-19 pandemic; however, by June and July 2020, it returned to the regular level. While Figure 1 is for the total number of transplants for all race types by organ types (living vs. deceased), Figure 2 disaggregates the effect by race groups. Figure 3 and Figure 4 further disaggregate by living and deceased donors by race. The disaggregated results in Figure 2 reveal that the decline in the number of kidney transplants due to COVID-19 shock was driven mainly by the decrease in White candidates’ transplantation. Further, Figure 3 and Figure 4 together demonstrate that the disproportionate negative impact on White transplant candidates stems from the decline in the living donor, as opposed to a deceased donor, transplants. Figure 5, Figure 6 and Figure 7 illustrate the transplant change rate between the pre-pandemic (December 2019–February 2020) and the early pandemic (March 2020–May 2020). The three figures show a similar pattern that states in the Northeast, Northwest, and Midwest regions have a higher reduction in the transplant rate than other states.

The short-term effect analysis included 106,300 waitlisted candidates registered, as well as 7159 deceased donor and 2297 living donor transplant recipients between 1 December 2019–29 February 2020. The number of the transplant candidates who were removed from the waitlist due to severe sickness or death was 4595 during the same period. The total number of transplant programs was 252.

Table 1 and Table 2 illustrate candidate characteristics in the pre-pandemic period (1 December 2019–29 February 2020) and during the pandemic period (1 March 2020–31 May 2020). The shares of White and minority candidates on the waitlist were similar prior to and during the pandemic: In the pre-pandemic period, the waitlist included 98,534 candidates. Of these, 35,768 (36%) were White while 62,766 (64%) were minority candidates. During the pandemic period, 98,594 candidates were enrolled on the waitlist. Of which 35,444 (36%) were White while 63,150 (64%) were minority candidates. The bivariate analyses also demonstrated similar results between the two periods.

In the pre-pandemic period (Table 1), White candidates were older than minority candidates (58 vs. 54, *p* < 0.01). White candidates had a statistically significantly higher percentage of male candidates (63%) compared to minority candidates (61%). There was a statistically significant difference in insurance types between White and minority candidates (*p* < 0.01). The majority of White candidates used private insurance (50%) to pay for their health care expenses, and the following most common payment methods among White candidates were Medicare (40%), Medicaid (6%), and other insurance (4%). By contrast, most minority candidates had Medicare (44%), and the next common payment methods were private insurance (39%), Medicaid (14%), and other insurance (3%).

There were statistically significant differences in CPRA and KDPI between White and minority candidates. Minority candidates had a higher mean CPRA at 23.69 compared to 18.62 in the White candidates. However, White candidates had a slightly higher mean KDPI than minority candidates (0.48 vs. 0.47, *p* < 0.01). In terms of the candidate transplant status, White candidates had a significantly higher percentage of transplants than minority candidates (6.86 vs. 4.73, *p* < 0.01), yet White candidates had a significantly greater percentage of removals (sickness or deaths) compared to minority candidates (2.75 vs. 2.07, *p* < 0.01). There was a statistically significant difference in transplant center volume between White and minority candidates (2116 vs. 2952, <0.01).

Table 2 shows the results of an analogous analysis for during the pandemic. All results were similar to the pre-pandemic period, and both differences between the two groups and the statistical significance associated with the differences remained comparable.

Table 3 presents the changes in the candidate transplant status between pre-pandemic and pandemic periods by sex, race, insurance type, and geographic regions. In general, during the observation period, the kidney transplant rate was reduced by 25.6%, and the removal rate was increased by 1.18%. The transplant rate declined more for female candidates than male (−26.33% vs. −25.14%). However, female candidates experienced the decreased rate of removal from the candidate list during the pandemic while male candidates experienced the increased rate of removal. (−10.74% vs. 8.46%). As for the racial variation, White candidates had a more significant transplant rate reduction than minority candidates (−32.19% vs. −20.15%) while minority candidates were found to have a higher removal rate than White candidates (9.23% vs. 9.45%). In terms of insurance and geographic regions, Medicaid candidates had a 36.42% increase in the removal rate. Candidates in the Northwest region of the United States had a more significant reduction in the transplant rate and a greater increase in the removal rate during the pandemic period relative to the pre-pandemic period.

### 3.2. Competing Risk Analysis

Table 4 and Table 5 present the result of the competing risk analysis for the candidate transplant status, waitlist, transplants, or removals due to sick/death. As noted in the Methods section, when transplant patients were identified with events of interest, sick/death patients were treated as a competing event (Table 4). When the events of interest were sick/death patients, the competing event is transplant patients (Table 5). The competing risk analysis uses a sub-distribution hazard ratio (SHR) to evaluate the risk of an event happening in a population over a certain period. It is determined by comparing the rate of the event in one group to the rate of the event in another group, allowing us to determine the likelihood of the event occurring in one group relative to the other. This comparison helps us to understand the relative change in the instantaneous rate of the occurrence of the event in those subjects who are event-free or who have experienced a competing event.

In Table 4, to investigate the differential effect of the pandemic by race on time-to-transplants we interacted race with the stage of pandemic period. In general, White candidates revealed a shorter waiting time to receive transplants than minority candidates. Compared to the pre-pandemic period, there was a great reduction in transplant waiting time during the pandemic period. Specifically, during the pre-pandemic period (i.e., pandemic period = 0), White candidates experienced a shorter transplant waiting time than minority candidates, and the sub-distribution hazard ratio of the transplant waiting time was decreased by 32% (*p* < 0.01). During the pandemic period, White candidates were even quicker to receive transplants. Compared to minority candidates, White candidates’ sub-distribution hazard ratio of the transplant waiting time was reduced by 55% (SHR = exp(log(1.3195) + log(1.2065) + log(0.9733)) = 1.5495; *p* < 0.01). Furthermore, older patients waited a longer time to receive transplants (SHR = 0.99, *p* < 0.01) (Table 4) but a shorter time to sick/death (SHP = 1.04, *p* < 0.01) (Table 5). There were statistically significant differences among insurance types. Patients with Medicare had a shorter waiting time to receive transplant than patients with private insurance (SHR = 1.07, *p* < 0.01). However, patients with Medicaid or other insurance waited longer to receive transplants, compared to patients with private insurance. In addition, the model shows that patients with high CPRA levels waited longer to receive compatible donor organs (SRH = 0.9997, *p* < 0.01). When patients were registered in a transplant center with high-volume candidates, the sub-distribution hazard ratio of the transplant waiting time was increased by 0.04% (*p* < 0.01). Finally, patients experienced a longer waiting time when their registration centers were in a county where high numbers of COVID-19 deaths were observed (SHR < 0.9999, *p* < 0.01).

In Table 5, the findings reveal that White candidates had a higher risk of being removed from the waitlist due to severe sickness or death, compared to minority candidates. There was also a great reduction in sick/death removal time during the pandemic period. Analysis interacted race with the stage of pandemic period and found the following: During the pre-pandemic period (i.e., pandemic period = 0), White candidates’ sub-distribution hazard ratio of the removal risk was increased by 16% (*p* < 0.01). During the pandemic period (i.e., pandemic period = 1), White candidates had a higher removal risk than the pre-pandemic period, and the sub-distribution hazard ratio of the removal risk was increased by 25% (SHR = exp(log(1.1564) + log(1.1063) + log(0.9749)) = 1.2472; *p* < 0.01). Older, male, Medicare, high CPRA patients had a statistically significant higher risk of removals due to severe sickness or death. Patients who were enrolled in a transplant center with high-volume candidates were expected to have a lower risk of removals (SHR = 0.9999, *p* < 0.01). The number of COVID deaths at the county level increased the risk of removals by 0.01% (*p* < 0.01).

Our missing rate in the competing risk analysis was 0.078%, which was only due to CPRA. We conducted tests to determine if the missingness was associated with other variables and found that White, female, and younger candidates were more likely to have missing values in CPRA. To ensure robustness, we removed CPRA from the competing risk analysis and the results showed that all the *p* values are similar to the competing risk models in Table 4 and Table 5, and the significant coefficients still had the same direction of association. We understand that we cannot completely eliminate any potential bias caused by the missingness, but we believe the effect is minimal and can be ignored.

## 4. Discussion

This study comprehensively examines the effects of the COVID-19 pandemic on patient kidney transplantation status on waitlist, transplantation, and waitlist removal due to illness or death rates across sociodemographic groups, including race, insurance groups, sex, and geographic region, with several important findings.

First, the number of transplants decreased to 3084 during the pandemic period from the pre-pandemic number of 5422. This finding is understandable and well-documented in the literature as most transplant centers in the United States either ceased or reduced the number of transplants being performed during the initial pandemic period [11,12,13]. Interestingly, the mean center transplant volume increased for both White (2115.93 to 2335.01) and minority patients (2951.54 to 3195.95) during the pandemic period. It is understandable that this number may be increased during the overall pandemic period as transplant centers attempt to adjust from the immediate post-pandemic slowdown period previously mentioned by increasing transplant volumes to accommodate more patients on the waiting list. This would seemingly demonstrate a remarkable recovery and subsequent progression of the United States’ organ transplantation network over the course of the pandemic period.

From a geographic standpoint, the Northeast had the greatest decrease in transplant volume at −47.69% and the largest increase in sickness or death at 45.81%. The change in geographic transplant volume could be considered in various factors of the pandemic, including geographic COVID-19 related restrictions. Interestingly, the Northeast was considered among one of the stricter regions in terms of COVID-19 related restrictions, with 9 states from this region (Vermont, District of Columbia, Delaware, Virginia, New York, Maine, Connecticut, Rhode Island, and New Jersey) being considered among the top 10 states with the most coronavirus restrictions [14]. Conversely, the South, which had the smallest decrease in transplant volume at −17.77% and the second highest decrease in sickness or death at −13.28% had four states (Florida, Texas, South Carolina, and Mississippi) considered among the top 10 states with the least coronavirus restrictions. While higher coronavirus restrictions do not directly mean decreased transplants, they may point to a certain region’s likelihood of adjusting transplant proceedings in response to the pandemic.

With respect to the competing risk analysis, one of the most significant findings was the reduction in transplant waiting times for White versus minority patients. Pre-pandemic, it was shown that White patients already waited 32% less time (*p* < 0.01) for their transplants compared to their minority counterparts. This study showed that Whites not only continued to have shorter wait times during the pandemic period, but also their wait times actually decreased further to waiting about 55% (*p* < 0.01) less time than their minority counterparts. This highlights the exacerbation of racial disparities in renal transplant waitlist times in the United States. This long-standing racial disparity for waitlist times in the United States has been well documented and further research is imperative to elucidate the cause of the established gap in care and of the pandemic-related widening of the disparity [15,16].

It was also noted that older patients spent a longer period of time on the waiting list (SHR = 0.99, *p* < 0.01). This is a known phenomenon and much of the literature relates older patients spending a longer period on the waiting list to decreased medical suitability compared to their younger counterparts [17]. In essence, increased testing, clearance, and overall management of comorbid medical conditions led these patients to experience more time on the waiting list.

This study found that Medicare patients waited significantly less time for their kidney transplants compared to patients with private insurance or Medicaid. While the exact cause of this discrepancy is not well understood, it is reasonable to presume this is partially because Medicare comprehensively covers the cost of kidney transplantation and associated costs. Through Medicare parts A, B, and D, any patient over the age of 65 is insured for every aspect of their kidney transplant procedure along with pre- and post-op care. While Medicaid and private insurances may also afford near-complete coverage in many instances, aspects, such as the approval speed and co-pays of these insurances, are not uniform and may play into the longer time spent on the waiting list for these patients, a disparity that has been documented previously [18].

Moreover, this study found that patients experienced a longer waiting time when their registration centers were in a county where high numbers of COVID-19 deaths were observed. It is reasonable to assume that in counties more overwhelmed with COVID-19, increased resources (staff, finances, PPE, etc.) in hospitals and transplant centers were allocated to units treating COVID-19-related cases. In these counties, there was likely a slower reopening of full transplant capacity leading to a longer time on the waitlist for many patients.

It is important to note that national organizations, such as UNOS and OPTN, have taken steps in the past to mitigate sociodemographic disparities in renal transplant. The kidney allocation system (KAS), which was recently revised in 2014, has made great strides in making the allocation of kidneys more equitable. The KAS incorporated multiple inclusive changes, such as allowing for patients to accrue waitlist time from dialysis initiation instead of from transplant referral, which had previously been a barrier to racial and gender minorities as these groups were often referred later than their counterparts [19]. More recently, ongoing discussion and change that have been occurring are the decreased utilization of race-based eGFR measurements and transitioning to eGFR universal for every race [20]. Currently, Black patients are given a higher eGFR value due to the race-based coefficients. Simply removing the race-based eGFR measurements would increase the prevalence of CKD among Black patients and also the severity classification of patients with existing kidney disease, thus potentially providing Black patients with more resources to match their disease severity, such as transplantation [21]. While national organizations have made significant improvements in the past and have existing initiatives committed to addressing disparities in transplantation, our study shows that sociodemographic inequities in transplantation are not fully addressed.

Lastly, although this paper is similar to a quasi-natural experimental design, we should be cautious to claim any causal evidence because COVID-19 led to substantial change in many sectors of healthcare (transplant, public health, healthcare administration, etc.). Due to the massive impact of the pandemic, many sectors simultaneously initiated new policies and operated at a different capacity than before the pandemic. Thus, at times, it is difficult to differentiate between the impact of the COVID-19 pandemic itself and the change in subsequent policies. However, considering the results of this study is important as the world moves past the COVID-19 pandemic, and the transplant network (UNOS, transplant centers, transplant surgeons, etc.) returns to baseline operation. The future outlook of sociodemographic considerations in waitlist status and transplantation as a whole is dependent on the transplant network recognizing these disparities and acting at various levels to combat any existing inequalities in care. Thus, there may be several implications for policy and future research. Further research should be conducted to elucidate the relationship between sociodemographic status and transplant waitlist status. In addition, studies are needed to determine what impact these sociodemographic disparities have on medical outcomes in this patient population. Policymakers at an institutional and national level should consider the results of this study to determine how disparities in transplant may be most efficiently addressed.

## 5. Conclusions

Based on this study, waitlist status and disposition varied significantly based on patient sociodemographic factors. During the pandemic period, minority patients, those with public insurance, older patients, and those in counties with high numbers of COVID-19 deaths experienced longer wait times. In contrast, older, White, male, Medicare, and high CPRA patients had a statistically significant higher risk of waitlist removal due to severe sickness or death. The results of this study should be considered carefully as we approach a rebounding transplantation network post-COVID-19, and further studies should be conducted to elucidate the relationship between a transplant candidate’s sociodemographic status and medical outcomes during this era.

## Figures and Tables

**Table 1:** Descriptive Statistics (Pre-pandemic: December 2019–February 2020).

	White (*n* = 35,768)		Minority (*n* = 62,766)		Cohen’s d ^d^/ Cramér’s V ^v^	*p*-Value
Age, mean (sd)	57.82	(13.43)	54.15	(12.87)	−0.28 ^d^	<0.01
Male, *n* (%)	22,598	(63.18)	38,404	(61.19)	0.02 ^v^	<0.01
Primary insurance						
Private insurance, *n* (%)	17,938	(50.15)	24,677	(39.32)		
Medicaid, *n* (%)	2048	(5.73)	8541	(13.61)	0.14 ^v^	<0.01
Medicare, *n* (%)	14,251	(39.84)	27,424	(43.69)		
Other insurance, *n* (%)	1531	(4.28)	2124	(3.38)		
CPRA ^+^, mean (sd)	18.62	(32.47)	23.69	(35.65)	0.15 ^d^	<0.01
KDPI ^+^, mean (sd)	0.48	(0.26)	0.47	(0.26)	−0.04 ^d^	<0.01
Transplant, *n* (%)	2454	(6.86)	2968	(4.73)	0.05 ^v^	<0.01
Sick/death, *n* (%)	984	(2.75)	1300	(2.07)	0.02 ^v^	<0.01
Center transplant volume, mean (sd)	2115.93	(2291.85)	2951.54	(3013.25)	0.30 ^d^	<0.01

^+^ CPRA: pre-pandemic White *n* = 35,696; minority *n* = 62,762; KDPI: pre-pandemic White *n* = 6658; minority *n* = 11,608.

**Table 2:** Descriptive Statistics (Pandemic: March 2021–May 2021).

	White (*n* = 35,444)		Minority (*n* = 63,150)		Cohen’s d ^d^ / Cramér’s V ^v^	*p*-Value
Age, mean (sd)	57.52	(13.54)	53.90	(12.93)	−0.28 ^d^	<0.01
Male, *n* (%)	22,420	(63.25)	38,726	(61.32)	0.02 ^v^	<0.01
Primary insurance						
Private insurance, *n* (%)	17,746	(50.07)	24,904	(39.44)		
Medicaid, *n* (%)	2055	(5.80)	8721	(13.81)	0.14 ^v^	<0.01
Medicare, *n* (%)	14,076	(39.71)	27,378	(43.35)		
Other insurance, *n* (%)	1567	(4.42)	2147	(3.40)		
CPRA ^+^, mean (sd)	18.46	(32.35)	23.52	(35.54)	0.15 ^d^	<0.01
KDPI ^+^, mean (sd)	0.47	(0.26)	0.46	(0.26)	−0.04 ^d^	<0.01
Transplant, *n* (%)	1664	(4.69)	2370	(3.75)	0.02 ^v^	<0.01
Sick/death, *n* (%)	891	(2.51)	1420	(2.25)	0.01 ^v^	<0.01
center transplant volume, mean (sd)	2335.01	(2370.08)	3195.95	(3094.66)	0.30 ^d^	<0.01

^+^ CPRA: pandemic White *n* = 35,375; minority *n* = 63,145; KDPI: pandemic White *n* = 5868; minority *n* = 10,193.

**Table 3:** Change in Candidate Transplant Status.

		Transplant	Sick/Death	Waitlist
Total		−25.60%	1.18%	1.56%
Sex	Female	−26.33%	−10.74%	1.61%
	Male	−25.14%	8.46%	1.54%
Race	Minority	−20.15%	9.23%	1.47%
	White	−32.19%	−9.45%	1.73%
Primary insurance	Private insurance	−26.04%	3.22%	1.62%
	Medicaid	−25.18%	36.42%	2.33%
	Medicare	−24.76%	−3.84%	1.12%
	Other insurance	−32.70%	−12.09%	3.58%
Geographic region	Midwest	−20.49%	7.37%	0.38%
	Northeast	−47.69%	45.81%	1.12%
	South	−17.77%	−13.28%	2.10%
	West	−25.37%	−14.73%	1.88%

**Table 4:** Estimate of Time-to-Transplant.

	SHR	S.E.	95% C.I.		*p* Value
White	1.3195	0.0354	1.2520	1.3907	<0.01
Pandemic period	1.2065	0.0162	1.1752	1.2387	<0.01
White Pandemic period	0.9733	0.0086	0.9566	0.9903	<0.01
Age	0.9894	0.0008	0.9880	0.9909	<0.01
Male	0.9624	0.0206	0.9229	1.0037	0.07
Private insurance (base)					
Medicaid	0.7069	0.0286	0.6530	0.7653	<0.01
Medicare	1.0707	0.0235	1.0256	1.1179	<0.01
Other insurance	0.6560	0.0417	0.5792	0.7430	<0.01
CPRA	0.9976	0.0003	0.9970	0.9982	<0.01
Center transplant volume	0.9996	0.0000	0.9996	0.9997	<0.01
County COVID-19 death	0.9999	0.0000	0.9999	0.9999	<0.01

**Table 5:** Estimate of Time-to-Sick/Death.

	SHR	S.E.	95% C.I.		*p* Value
White	1.1564	0.0496	1.0631	1.2578	<0.01
Pandemic period	1.1063	0.0123	1.0824	1.1307	<0.01
White pandemic period	0.9749	0.0121	0.9515	0.9990	0.04
Age	1.0376	0.0015	1.0347	1.0405	<0.01
Male	1.1114	0.0356	1.0437	1.1833	<0.01
Private insurance (base)					
Medicaid	1.1096	0.0636	0.9918	1.2415	0.07
Medicare	1.2828	0.0429	1.2015	1.3697	<0.01
Other insurance	1.1387	0.0917	0.9725	1.3334	0.11
CPRA	1.0017	0.0005	1.0008	1.0026	<0.01
Center transplant volume	0.9999	0.0000	0.9999	1.0000	<0.01
County COVID-19 death	1.0001	0.0000	1.0001	1.0001	<0.01

## Data Availability

Publicly available datasets from UNOS STAR were analyzed in this study. This data is available for request here: https://optn.transplant.hrsa.gov/data/view-data-reports/request-data/.
